# Comparative resilience and precision of digitized optical counting using ImageJ during routine mosquito (Diptera: Culicidae) sample processing

**DOI:** 10.1093/jisesa/ieaf026

**Published:** 2025-03-14

**Authors:** Ayla Faraji, Kelsey A Fairbanks, Ary Faraji, Christopher S Bibbs

**Affiliations:** Salt Lake City Mosquito Abatement District, Salt Lake City, UT, USA; Salt Lake City Mosquito Abatement District, Salt Lake City, UT, USA; Salt Lake City Mosquito Abatement District, Salt Lake City, UT, USA; Salt Lake City Mosquito Abatement District, Salt Lake City, UT, USA

**Keywords:** identification, operation, trap, vector, efficiency

## Abstract

Surveillance is integral for the targeted and effective function of integrated vector management. However, the scale of surveillance efforts can be prohibitive on manpower, given the large number of traps set, collected, processed, and enumerated. For many public health agencies, the sheer effort of weekly trapping, combined with the processing of numerous traps, is a major capacity challenge. To reduce employee fatigue and increase throughput, estimation methods are used in a diagnostic capacity to determine threshold numbers of mosquitoes (Diptera: Culicidae) for operational decision-making. Historically, volume and mass measures correlated to a known number of mosquitoes are the oldest and most widely used within mosquito control programs. Image processing methods using digital counting software, such as ImageJ, have not been tested rigorously in the context of high throughput usage experienced in mosquito operations. We stress-tested volume, mass, and image processing methods using sample calibrations from early in the year and applied them throughout a mosquito active season. We additionally tested resilience with samples that had been frozen, desiccated, old, or from an excessively large trap collection. Furthermore, we compared magnitudes of error after intentionally deviating from best practices. In all cases, mass and volume encountered significant errors. In contrast, the digitized-optical counting method was resilient to going long periods of use without recalibrating, handling different species compositions, and processing aged or damaged samples. If a program has limited logistical power, the aforementioned image-processing method confers the best balance of accuracy and expediency for time-sensitive workloads and efficient operational decision making.

## Resumen

La vigilancia es funda mental para el funcionamiento específico y eficaz de los sistemas integrados de vigilancia de vectores gestión. Sin embargo, la escala de los esfuerzos de vigilancia puede ser prohibitiva en términos de personal, una gran cantidad de trampas colocadas, recolectadas, procesadas y enumeradas. Para muchas agencias de salud pública, el gran esfuerzo de captura semanal, combinado con el procesamiento de trampas numerosas, es un gran desafío de capacidad. Para reducir la fatiga de los empleados y aumentar el rendimiento, se utilizan métodos de estimación en una capacidad diagnóstico para determinar el número umbral de mosquitos (Diptera: Culicidae) para la toma de decisiones operativas. Históricamente, las medidas de volumen y masa correlacionados con un número conocido de mosquitos son los más antiguos y los más utilizados dentro programas de control de mosquitos. Métodos de procesamiento de imágenes mediante software de conteo digital, como ImageJ, no se han probado rigurosamente en el contexto de uso de alto rendimiento experimentado en operaciones con mosquitos. Probamos métodos de volumen, masa y procesamiento de imágenes, utilizando calibraciones de muestra del principio del año y se aplicaron durante toda la temporada de actividad de los mosquitos. También probamos la resiliencia con muestras que habían sido congeladas, desecadas, viejas o de una colección de trampas excesivamente grande. Además, comparamos las magnitudes de error después de desviarse intencionalmente de las prácticas mejores. En todos los casos, la masa y el volumen se encontraron niveles de error significativos. Por el contrario, el método de conteo óptico digitalizado fue resistente a períodos largos de uso sin recalibrar, uso con composiciones de especies diferentes y procesar viejas o dañadas muestras. Si un programa tiene un poder logístico limitado, el método de procesamiento de imágenes antes mencionado ofrece el mejor equilibrio entre precisión y conveniencia para cargas de trabajo sensibles al tiempo y toma de decisiones operativas eficientes.

## Introduction

Surveillance is a vital process of longitudinal data collection (Petrić et al. 2014, Aryaprema et al. 2023) that establishes an empirical understanding of mosquito abundance, diversity, and dispersion characteristics (Yang et al. 2009, Chen et al. 2011, Drakou et al. 2020). Time-sensitive data of sufficient density is required to estimate risk and plan interventions to protect public health (Farajollahi et al. 2009, Yang et al. 2009, Drakou et al. 2020). The urgency of decision-making is further amplified when dealing with rapidly proliferating mosquito populations (Crepeau et al. 2013, Drakou et al. 2020) and outbreaks of mosquito-borne pathogens, particularly West Nile virus from *Culex* spp. and imported cases such as dengue or Zika virus that can be vectored by invasive *Aedes* spp. (Connelly et al. 2020, Yee et al. 2022). Ultimately, this requires dealing with large volumes of sample material and having good standard operating procedures that allow for data collections to remain consistent over time (Reisen et al. 1983, 2000, Unlu and Farajollahi 2012, Jaworski et al. 2019). Unfortunately, a program can quickly experience stress fractures in operational protocols at the scale needed for public health vector surveillance (Farajollahi et al. 2009, Chen et al. 2011, Crepeau et al. 2013).

Using the Salt Lake City Mosquito Abatement District (SLCMAD) as an example, monitoring of roughly 285 km^2^ in high elevation, arid, sagebrush floodplains approaching the Great Salt Lake is conducted twice-weekly at over 40 surveillance sites. If data are enumerated same-day for the dates of trap collection, this only affords a half-day at best for sorting, counting, and speciation to inform management decisions during the subsequent evening of operations. The reality is that limited capacity, even in well-established programs (Crepeau et al. 2013, Aryaprema et al. 2023), forfeits the ideal of exact, fast, and consistent data. Implementing vetted diagnostic protocols compromises on this pitfall by reducing the burden of the workload, and improving the rapidity and cost-effectiveness of data collection (Jaworksi et al. 2019). For example, sub-sampling total collections for speciation and then extrapolating that count on species data can still allow reliable inference on mosquito bionomics as long as there is an understanding of sources of error (Jaworski et al. 2019).

Some common methods for rapidly enumerating mosquito collections include taking mass readings (Steiger et al. 2016), using volumetric measurements (Smith et al. 2010), extrapolating off surface area (Rey et al. 2006), image processing to acquire a semi-automated optical count (Mains et al. 2008, Kesavaraju and Dickson 2012), and even hybrid methods combining multiple approaches (Henderson et al. 2006, Schäfer et al. 2008). However, humidity fluctuations, environmental desiccation, and mosquito population age structure can jeopardize the reliability of mass readings (Kesavaraju and Dickson 2012). While differential body size, either because of species differences or rearing factors (Schneider et al. 2011), can also skew both volumetric and mass-based estimations.

There have been excellent analyses vetting the error rates of these methods across conventional use (Jaworski et al. 2019). However, the error rates within any of these protocols are recursive to the end-user and sample quality. There are several notable risks that compromise the data and invalidate longitudinal, or even cross-agency, comparison. Are estimations consistent across the physical condition of the sample? Do they skew with varying species composition? Does the error amplify within singularly large trap collections? If a program had limited logistical power, which method confers the best balance of accuracy and throughput? In an effort to build upon existing work comparing estimation methods (Jaworski et al. 2019), we performed an operationally relevant assessment on the resilience of mass, volumetric, and optical-counting methods against non-ideal sample conditions.

## Materials and Methods

**Fig. 1.**
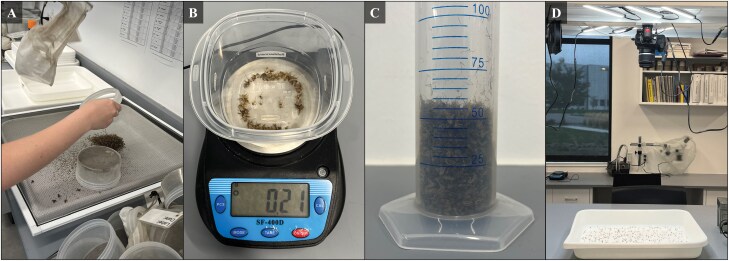
Processing methods used in study, to include (A) universally filtering and cleaning up samples to be mosquito-exclusive. (B) Mass estimations and (C) volumetric estimations were compared to (D) a digitized optical-counting method using ImageJ with high-contrast pictures.

Mosquitoes were collected during 24-h trap cycles twice a week using pressure-regulated CO_2_ baited at 300 ml/min, or ~50 g/h of the mass of liquid CO_2_ loaded into the cylinders, and CDC-style 3D printed Salt Lake City Traps (SLCT) (Bibbs et al. 2024). Collections ranged across an established surveillance network, spanning urban/residential tracts, rural wetland, and industrial transition zones. The 26-wk data collection period of twice-weekly trapping was split into the first 13 wk and latter 13 wk due to species composition shifts in the area. A total of 72 trapping events were used for assessments, with a pooled mosquito total of over 520,000 individual mosquitoes used in the study. Upon return to the laboratory for processing, mosquitoes were chilled at −80 °C for 20 min and then filtered over a fiberglass window screen (Clear Advantage, Saint-Gobain ADFORS, Grand Island, NY) to allow pass through of biting midges and other small non-target insects (Fig. 1A). Large non-targets, such as Chironomid midges and large-bodied flies, were removed by hand. Cleaned samples were then set aside for same-day processing with one of 4 handling methods.

Traps used for the study were initially hand counted and identified to species using morphological keys (Darsie and Ward 2005) to obtain the entire species composition and abundance. This value was used as the absolute numerical target for the other estimation methods to determine error. Volumetric measurements were taken in a 250-ml graduated cylinder in a maximum up to no more than 90% of the total volume of the cylinder. Cylinders were gently tapped 5 times on the counter-top to settle the contents for measurement. The measure was taken with mosquito bodies, not wings or legs, clearing the measure line (Fig. 1B). For mass measurements, a plastic weigh boat was tared on a digital scale (Horizon SF-400D, Westlake Tactical, Bridgeville, PA) and filled no more than half the total volume with mosquitoes. The mass reading, in grams, was recorded for the contents (Fig. 1C). In both volumetric and mass measurements, the process was repeated 3 times each, with the designated score for that trap being the average of the 3 readings. If more than one mean measurement was required to exhaust the number of mosquitoes, then the means were summed for the final value.

For the digitized counting system, a computer was connected to a digital SLR camera (EOS Rebel T1i, Canon U.S.A., Inc., Huntington, NY) with a standard range of 18 to 55 mm lens set to 34 mm optical zoom. The face of the lens was suspended 73.5 cm above the benchtop (Fig. 1D). The bench was illuminated using a 6-piece LED string light setup (12-inch bars at 31 watts, Litever, Guangdong, China) casting 2,000 lumens at a color temperature of 5,000 K. The camera was operated with on-board live view shoot software that was pre-installed from the camera. Trap contents were emptied onto a white photo-developing tray (20.32 × 3 × 25.4 cm; CescoH; B&HPhoto Video, New York City, NY) and centered in the frame with no black space visible from the digital viewer. Mosquitoes were evenly distributed across the viewing frame to create as many breaks in contrast around individual mosquitoes as possible; for the size of the tray, this was best at around 30 to 45 ml of mosquitoes spread around the tray. The final count was summed from the collective of all required pictures to exhaust the trap contents, which was derived from ImageJ (Rasband, W.S. ImageJ, U.S. National Institutes of Health, Bethesda, MD) loaded with a macro following the processes of Mains et al. (2008) with the adaptations by Kesavaraju and Dickson (2012):

*run(“8-bit”);*

*setThreshold(0, 50);*

*run(“Convert to Mask”);*

*run(“Analyze Particles…” , “size=38-infinity circularity=0.00-1.00 show=Nothing exclude clear summarize”);*

*close();*

Each method was calibrated at the beginning and end of the 26-wk study using the naturally occurring trap contents from SLCMAD. This was derived from 3 trap sites collecting ~1,000 mosquitoes, 3 sites collecting ~2,000 mosquitoes, and 3 final sites collecting ~5,000 mosquitoes per collection event. A mean numerical value was correlated per ml (volumetric) or per gram (mass) using the hand counts and total volume/mass of the corresponding 1,000/2,000/5,000 mosquito samples. For the digital count, this functioned as a validation step of what the macro generated for body counts, whereby the deviation from the total hand count was enumerated. However, the camera system could not be recalibrated per se without changing the macro programming, which was not the focus of this study. The initial calibrations were used to estimate the collections of mosquitoes during the active season 6 times each in the former and latter 13-wk intervals, allowing natural changes to species composition to be a source of error. The values determined with the high throughput methods were verified against a hand count of the entire contents of the same trap. Error was then determined in the deviation from hand counts. The calibration process was repeated with the same number of trap collections and number of mosquitoes at the end of the study to compare to the first calibration.

### Resiliency Measures

For each method, the stress tolerance of each method was measured with specific clerical errors or deviations from an acceptable protocol that we expected to be commonplace. The logic stems from cultural/within-task decision making inherent to all of these methods that can change details of performance. Examples could be between experienced staff and trainees, or between naturally careful practitioners and rushed/careless practitioners. Trap contents used for this evaluation were from the beginning of season, chronologically when pre-season calibrations were made. Therefore, initial calibrations were used to infer errors from estimates versus actual counts. For volume, a series of 3 samples at 1,000 mosquitoes, 2,000 mosquitoes, and 5,000 mosquitoes were measured normally (3-point mean for each quantity) and then compared to the same procedure but with mosquitoes forcefully packed into the weigh boat in the entirety of the sample instead of naturally settling, even if it means taking the mass on a partial trap collection. For volumetric, the same sample sizes were used to take measures on a sample tapped 5 times versus a sample that was untapped and loose. For the camera, the viewer was overcrowded intentionally using batches of mosquitoes from 45 to 225 ml of mosquitoes (45-ml increments). Each 45-ml increment encapsulated ~1,000 mosquitoes, given the normal diversity, body size, and environment where this study was carried out. But that volume will result in more or less mosquitoes depending on body size. These were repeated across 3 trap sites. This was to determine the progressive error/loss of trust in the system as the image becomes over crowded. As before, all metrics were compared to verified hand counts of the same samples.

Additionally, mosquitoes of varying handling conditions were set aside from early season, when calibrations were made and expected to be most accurate. These groups were fresh collections within 30 min of processing from the field, mosquitoes stored in a household freezer for 5 d, mosquitoes stored in a household freezer for 60 d, mosquitoes allowed to desiccate on the bench top for 5 d, and mosquitoes allowed to desiccate on the benchtop for 60 d. This was to simulate non-ideal sample processing situations, such as during supplemental investigations that are conducted with less priority than operational surveillance or when work staff is limited. An additional single-point stress test was done on a one-night trap collection exceeding 35,000 mosquitoes. The single-trap burden was used to evaluate how error scales with outliers.

### Data Analysis

For all categories of testing, the dependent variable was the resulting mean percentage error of an estimation method as determined from a hand-count on the same sample. Calibrations for pre- and post- season were compared via paired *t*-test. A 2-way ANOVA with interaction was performed to analyze the effect bracketed 13-wk periods (independent variable 1) of the estimation method (independent variable 2) and on the mean percent error. Comparisons estimation method (independent variable 1) with fresh, frozen, desiccated, and large trap collection samples (independent variable 2) also were analyzed with 2-way ANOVA with interaction, whereby the effect of estimation method and sample condition was determined on the mean percent error of sample counts. Data collections on user-error protocol deviations were analyzed with 2-sample *t*-tests (Welch’s test) between the normal (group 1) and non-ideal condition (group 2) for volume and mass. The mean magnitude of error for overcrowded imaging conditions were analyzed with 1-way ANOVA/Tukey HSD, with discreet volumes ranging 45 to 225 ml (independent variable). All statistical analyses were conducted using R v. 4.2.0 (R Core Team 2022).

## Results

Initial calibrations of methods averaged out to 20.2 mosquitoes per milliliter and 509.5 mosquitoes per gram. Image processing was validated to be 1.4% error from actual counts. The mosquito composition of the trap collections used for these was proportionally biased towards *Aedes dorsalis* (Meigen) (Diptera: Culicidae) and *Culex tarsalis* Coquillett (Diptera: Culicidae), with some blended representation from *Culiseta inornata* (Williston) (Diptera: Culicidae), *Cx. erythrothorax* Dyar, and *Cx. pipiens* L. As the season progressed, water availability and season change drove down diversity and trap collections tended to be mostly monotypic for either *Ae. dorsalis*, *Cx. erythrothorax*, or *Cx. tarsalis*. During post season re-calibration, the numerical correlate for the estimation methods shifted significantly to 23.5 mosquitoes per milliliter (*t*_8_ = 4.58, *P* = 0.002) and 407.26 mosquitoes per gram (*t*_8_ = -5.37, *P* < 0.001). The digital counting system remained at 1.3% error.

**Fig. 2.**
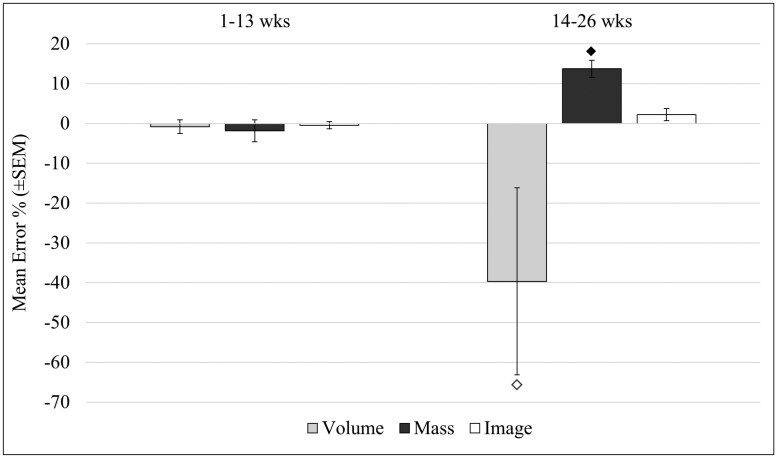
Comparisons of error magnitude when using pre-season calibrations of volume, mass, or digital estimation methods and applying them to wild collections from the field through a season interval of 1 to 13 wk (left) and 14 to 26 wk with the same set of calibrations. I-bars denote the standard error of the mean. Diamonds indicate significant increase in error over the prior 13-wk interval within the same method. Different symbols (hollow or filled) indicate separate pairwise analyses.

This early versus late season difference in the reliability of estimation methods was reflected in the seasonal trap counts using only the initial calibrated values. When grouping seasonal timeframes to 1 to 13-wk interval or the 14 to 26-wk interval, all methods had less than 2% total error in estimation from hand counts during the first block of the season (Fig. 2), since the calibrated measures were using the same mosquito compositions as was typical for the early block of the season. A significant interaction was found between the estimation method and the seasonal timeframe (*F*_2,30_ = 4.25, *P* = 0.024), with the estimation methods becoming more error prone in the latter half of the season. Generally, all methods tended to undercount a little by default. However, errors skewed dramatically with the seasonal progression when mixed collections started to become monotypic *Culex* spp. or *Aedes* spp. (*F*_2,30_ = 4.06, *P* = 0.028). The latter half of the season yielded significantly higher magnitudes of error (Fig. 2), with volumetric methods dramatically underestimating sample counts (*P* = 0.01) and mass significantly overestimating sample counts (*P* < 0.001). Although estimation error from the digital counting system averaged ~4% or less, it was consistent between the two halves of the seasonal collections.

**Fig. 3.**
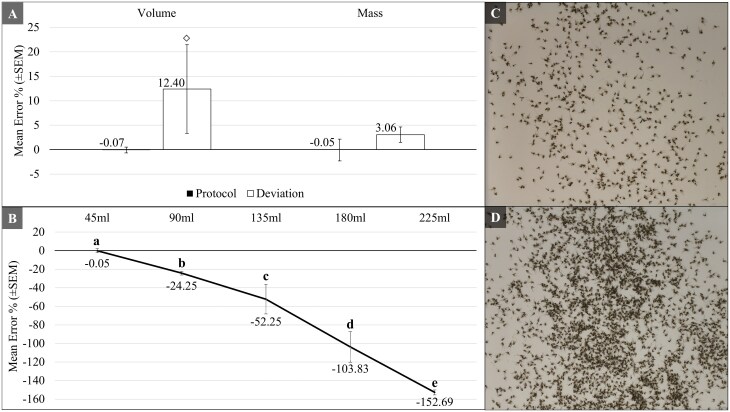
(A) Error was compared between normally executed and intentionally mishandled samples, using untapped volume, taking mass from an over-packed, compressed pile in a weigh boat (as opposed to loosely packed). Diamonds indicate significant increase in error as compared to data from a normally executed protocol. (B) Intentionally overcrowded image processor using escalating volumes of mosquitoes. Letters (a-e, low to high) denote significant differences in the amount of error between the volumes used for imaging. (C) Visual of an ideal image processing frame at 45 ml aliquot, corresponding to ~1,000 individual mosquitoes in this study. D) Visual of an overcrowded image processing frame from a 135 ml aliquot of mosquitoes, corresponding to > 3,000 individual mosquitoes in this study. I-bars denote standard error of the mean, with bars above or below the 0-axis representing overestimation or underestimation of mosquitoes, respectively.

When testing error from poor user implementation (Fig. 3A), mass measures were not significantly affected by overpacking the scale weigh boat (*t* = -1.36, df = 2.22, *P* = 0.295). Volume measures significantly overestimated the totals (*t* = 4.02, df = 2.15, *P* = 0.05) when mosquitoes were not tapped, since the measure would have been artificially occupying more space in the cylinder. For the digital counting system (Fig. 3B), error resulting in undercounting of mosquitoes essentially doubled in total error for every 45-ml of mosquitoes overcrowding the viewing frame (*F*_4,10_ = 34.87, *P* < 0.001). Predictably, each subsequent overload of the imaging system was significantly more error prone, with the progression of error from high error to low error reflecting 225 > 180 > 135 > 90 > 45 ml.

**Fig. 4.**
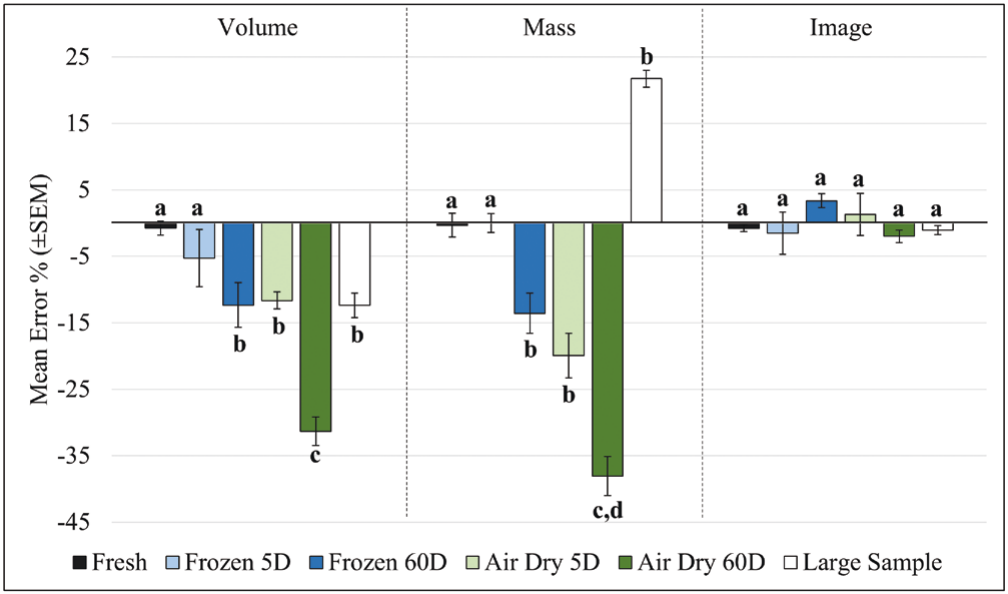
Non-ideal sample conditions were evaluated with each method, to include samples from cold storage, neglected samples that had air dried, and overabundance from singularly large trap collections. The error shown in the bar graphs was derived from comparing the deviation of the method from a hand count on the same sample. I-bars denote standard error of the mean for the bars shown. Letters indicate significant difference between groups based on absolute deviation from the 0-axis, with bars above or below the 0-axis representing overestimation or underestimation of mosquitoes, respectively.

**Fig. 5.**
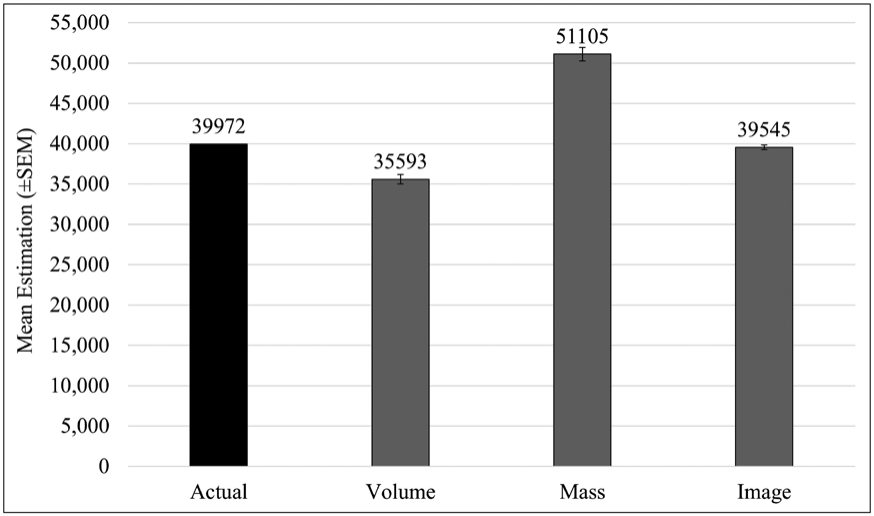
The effect size of the error from each method was visualized by showing the actual count of a large trap as determined by hand versus the mean estimation after 3 separate measurement instances for the respective methods on the x-axis. I-bars denote standard error of the mean for the bars shown.

When handling varying sample qualities (Fig. 4), estimation methods were increasingly more error-prone with declining sample qualities (*F*_10,36_ = 23.54, *P* < 0.001). The effect of sample degradation was more detrimental than necessarily the counting method (*F*_5,36_ = 48.93, *P* < 0.001), as fresh samples (*P* = 0.96) and those frozen within the last 5 days (*P* = 0.53) had an insignificant loss in accuracy. That said, the digitized system did not experience significant changes in error rate across any sample condition (Fig. 4), unlike with volume and mass (*F*_2,36_ = 41.85, *P* < 0.001). When handling 60-d frozen samples volume and mass had similar underestimation of counts (*P* = 0.0075). Air-dried samples more severely affected mass, with 5-d desiccated samples (*P* = 0.0045) and 60-d desiccated samples (*P* < 0.001) resulting in as much as 20% to 30% underestimation from actual counts (Fig. 4). The singularly large sample was 39,972 mosquitoes collected in June, with 96% of the contents comprised of *Ae. dorsalis*. In this case, volume was significantly underestimated (*P* < 0.001) and mass significantly overestimated sample size (*P* < 0.001) (Fig. 4). However, the scaling of their percentage of errors is also useful to consider (Fig. 5), with the mean of 3 repeated attempts on the whole sample returning volume (off by 2,000) and imaging counts (off by 400) more similar to the actual count. Meanwhile, the mass method overestimated the count by an additional 10,000 mosquitoes (Fig. 5).

## Discussion

The imaging platform using ImageJ was consistently resilient against changes in species composition, sample age, sample integrity, and large collection numbers. Our interpretation, based on the development by Kesavaraju and Dickson (2012); the capacity testing by Jaworski et al. (2019) in a disparate environment of the upper Rhine Valley, Germany; and our current results in the arid scrublands of the central United States, leads us to conclude that the image processing appears to be resilient to different species compositions and environmental conditions without explicitly modifying the code for the geography of usage. This is in part because ImageJ is not actually identifying the species; rather it is just taking a body count. Identification and taxonomic work still need to be conducted independently, which is a drawback of all the methods used in this study. This method only failed egregiously when the camera view was overcrowded, an expected outcome given the capacity of this process. This translates to a maximum processing speed for a large number of traps, since programs are inherently limited by how many pictures they have to take to exhaust sample workflow. Hypothetically, if it takes 1 min to take one picture of ~1,000 mosquitoes of average size, and the trap contents require 10 pictures, we estimate that a minimum time of 10 min may be dedicated to the entire trap.

In that regard, mass and volumetric methods can be the most time efficient in the short term, since measuring 100 to 5,000 + mosquitoes would effectively require the same amount of time per trap. However, both mass and volumetric methods were error prone in most situations, with slight variation in the merits. Deviations were worst with small-sized mosquitoes, like *Cx. erythrothorax*, since the early season calibrations would have included heavier-bodied species like *Ae. dorsalis*. When methods were recalibrated on the same traps from late season, they were back to within acceptable tolerances. As a result, it is essential in the protocol with the volumetric and mass methods to recalibrate your number correlations to milliliter or grams frequently during the season. Such concerns also pertain to long-term storage (freezing) before processing, desiccation stress from dry environments or neglect, or even simply changes in mosquito body sizes, as these will all skew volume and mass estimation methods. Therefore, programmatic use of mass or volumetric methods will ideally require working with fresh samples, preferably in storage no longer than 1 wk, since error already begins to show within 5 d. For a high throughput program, this should not hinder processing. However, there will be increased risk when retroactively counting a sample that was not initially prioritized, or recounting old samples if there may have been an error in the first data collection.

This discrepancy also highlights the need for broader awareness of how methods may interact with species-specific traits and environmental factors. For example, if a program were to be facing surveillance burden for *Aedes aegypti* (L.), they may need to have a much higher density of traps to accurately monitor potential risk (Coalson et al. 2023). Furthermore, phenotypic plasticity with rearing factors, particularly larval crowding, can yield wide ranges of adult body size within the same species (Janousek and Olson 2006, Schneider et al. 2011), which then manifest in geographically contingent surveillance networks (Janousek and Olson 2006, Chaiphongpachara et al. 2018). With the aforementioned concerns, pre- and post-season mosquito body size may not even be correlated, even when discussing the same species throughout the season. In addition, “hot spots” of variation may occur concurrently (Janousek and Olson 2006, Chaiphongpachara et al. 2018, Coalson et al. 2023), stressing the accuracy of various methods, particularly mass and volume, which may need to be recalibrated per trap on every individual collection event in such an extreme case. In these circumstances, a hybrid approach may be best, whereby the tendency for error from certain methods can ameliorate the total loss of accuracy that may be encountered by each method alone (Henderson et al. 2006, Janousek and Olson 2006, Schäfer et al. 2008).

However, not all methods can hybridize safely. Measuring on surface area has the same sources of error as with volume but just in 2 dimensions. Taking a mass reading from the mosquitoes used for surface area (Henderson et al. 2006) is layering the aforementioned confounders together. Digitally implemented optical-counting techniques are somewhat different from surface area calculations (Mains et al. 2008, Kesavaraju and Dickson 2012) and do not rely on mosquitoes occupying a given amount of space on a piece of paper (Henderson et al. 2006, Rey et al. 2006). Instead, they use visual contrast to identify individual mosquitoes for automated counting (Mains et al. 2008, Kesavaraju and Dickson 2012). Because of this, error rates can be lower across different compositions and time periods of mosquito collections. The digitized-optical counting method is correspondingly resilient to going long periods of use without the need for recalibration, since the parameters are defined by macro code. There is also an added benefit of being easier to reproduce and compare both internally and across agencies. Nevertheless, overcrowding is a realistic issue for the user and should be limited to 45 ml of mosquito bodies (regardless of species, condition, or environment), per image. In our study that corresponds to ~1,000 mosquitoes, but this correlated number will fluctuate as mosquito bodies get smaller or larger, respectively. Effectively, that determines that the image processing is most effective as a hybrid with volumetric methods controlling how crowded the pictures become.

An often-overlooked variable is that, when running an operational program, managers will encounter (or experience) poor motivation or have operators with low technical training. The natural variability in the end-user compliance within daily operations can unknowingly compromise your methods for mosquito surveillance. Our study does not examine all possible user-handling scenarios. Due to limitations in scope, we hope that decision-makers for operational programs will recognize that these are real concerns, and use our information from what we envisioned to be the easiest and most likely pitfalls where an operator may employ a “shortcut” to the protocol to save time and effort. The damage from error, such as not tapping down your mosquito bodies for volumetric measurement, extends most significantly to longitudinal data as being unreliable. But even in the short term, overestimating mosquito numbers may lead to more adulticide treatments than is required, resulting in increased cost and labor. Or, if underestimating mosquito counts, a program may not effectively and efficiently intervene when they should. When handling large collections, the error was magnified considerably, leading to misrepresenting counts by as much as ± 10,000 mosquitoes. We should also consider that this error was using relatively monotypic collections with simple changes during the season. But if working with sudden changes in diversity, such as floodwater mosquitoes emerging after storms (Kirik et al. 2021) or land use gradients (Young et al. 2021), a program may have wholly unreliable data across large land areas during critical decision-making periods for weather response or sudden anthropogenic changes.

The image processing protocol is not an exception to poor method execution. However, it does require less maintenance and is not prone to many naturally occurring sources of error for a technician. This allows for more effective hybridization of methods, such as being able to take a raw count from ImageJ and using a sub-sample within reasonable tolerances (Jaworski et al. 2019) to extrapolate species data. This is less confounded than using mass or volume because an actual numerical count has been performed by a computer. As opposed to the number itself being extrapolated (Henderson et al. 2006, Rey et al. 2006, Schäfer et al. 2008, Smith et al. 2010, Steiger et al. 2016) in addition to a secondary extrapolation by sub-sampling a representative proportion (Jaworski et al. 2019). In the future, it is possible artificial intelligence integration can improve the resolution of image processing methods to compensate for crowding issues and general inconsistency in body size. Furthermore, machine learning hybrids with sample counting functions might eliminate the need for the additional sub-sample in order to identify species with as high of efficiency as the counting alone. Even without these technological advancements, image processing to count for operational mosquito surveillance, as well as general exploration of hybridizing methods, could benefit from expanded use, replication, and reporting in many areas of the world to build consensus on efficient techniques.

To summarize in one place, all of the mass, volume, and image processing are accurate when sticking with fresh samples, or those only frozen for up to 5 d. However, both volume and mass break down in accuracy with severely desiccated samples and those from extended cold storage when they were not calibrated specifically on those sample conditions. Additionally, volume and mass should be recalibrated for their proxy of count whenever species composition shifts, or ideally for every sample count. Whereas the camera system does not necessitate changes to its macro when species composition, and corresponding morphological variation, changes. In contrast, the camera method is time-limited, with a per-picture processing time that may multiplicatively increase the delay to estimate large amounts of mosquitoes. More importantly, the operator cannot cut corners in that situation by simply adding multiplicatively more mosquitoes into the viewing frame for the camera, or else the system will severely underestimate the counts. Despite these drawbacks, the benefit of reduced maintenance throughout the year and the resilience to unpredictable sample conditions leads us to believe the digitized system is a reasonable compromise on time management and the long-term accuracy of data collection to improve public health protection efforts.
